# Wright's Shifting Balance Theory and the Diversification of Aposematic Signals

**DOI:** 10.1371/journal.pone.0034028

**Published:** 2012-03-28

**Authors:** Mathieu Chouteau, Bernard Angers

**Affiliations:** Group for Interuniversity Research in Limnology and Aquatic Environment (GRIL) and Department of Biological Sciences, University of Montreal, Montreal, Canada; The University of Queensland, St. Lucia, Australia

## Abstract

Despite accumulating evidence for selection within natural systems, the importance of random genetic drift opposing Wright's and Fisher's views of evolution continue to be a subject of controversy. The geographical diversification of aposematic signals appears to be a suitable system to assess the factors involved in the process of adaptation since both theories were independently proposed to explain this phenomenon. In the present study, the effects of drift and selection were assessed from population genetics and predation experiments on poison-dart frogs, *Ranitomaya imitator*, of Northern Peru. We specifically focus on the transient zone between two distinct aposematic signals. In contrast to regions where high predation maintains a monomorphic aposematic signal, the transient zones are characterized by lowered selection and a high phenotypic diversity. As a result, the diversification of phenotypes may occur via genetic drift without a significant loss of fitness. These new phenotypes may then colonize alternative habitats if successfully recognized and avoided by predators. This study highlights the interplay between drift and selection as determinant processes in the adaptive diversification of aposematic signals. Results are consistent with the expectations of the Wright's shifting balance theory and represent, to our knowledge, the first empirical demonstration of this highly contested theory in a natural system.

## Introduction

Understanding how populations adapt to different selective environments is a central theme in evolutionary biology. Fisher's large population size theory (LST) [1] and Wright's shifting balance theory (SBT) [2], [3] of evolution were developed to explain the process of adaptation. In both theories, natural selection is the only evolutionary force capable of producing adaptation. However, they differ in the role accorded to neutral evolutionary processes [4], [5]. In Fisher's LST, only mutations generate the diversity on which selection acts, while in Wright's SBT, the random variation of alleles frequencies by genetic drift is a decisive factor in finding adaptive opportunities [5]. Accumulating evidence for adaptation by Fisher's LST has been reported in natural systems [4], [6] but adaptation via Wright's SBT has, to our knowledge, only been demonstrated under laboratory conditions [7]. Due to the difficulty in demonstrating the entire SBT process within a single system, especially in natural populations, this theory is still highly criticized [4], [6], [8].

Aposematic signals are one of the better understood examples of how natural selection can maintain stable phenotypes. Individuals that deviate from the predators' recognized signal will suffer increased predation and this frequency-dependent selection should promote uniformity [9], [10]. Paradoxically, impressive spatial variation in aposematic signals has been documented among populations of a variety of organisms [11]–[17]. Numerous models, including either the LST [18] and the SBT [19]–[22] theories as central mechanisms, have been suggested to explain the origin of such spatially structured variation [23]–[26].

Geographical variation in aposematic signals are frequent in the poison-dart frogs of Northern Peru [15], [27]–[30]. For instance, *Ranitomeya imitator* displays highly variable and distinct warning signals among different localities, even at the scale of a few kilometers [15], [31]. Previous studies have confirmed the role of predators in maintaining the geographic organization of aposematic signals in this species, whereas different predator communities perform localized homogenizing selection on distinct aposematic signals [32]. Nevertheless, between some regions of aposematic uniformity are zones characterized by a striking diversity of warning signals; a situation not expected in aposematic systems [9], [33]–[36].

The aim of this study is to assess whether genetic drift is a determinant process in the diversification of aposematic signals. The transient zone between two geographically and phenotypically distinct populations of *R. imitator* provides an exceptional natural system to conduct such a study, as selective pressures are expected to change drastically. The effects of drift and natural selection on phenotypic divergence were assessed using population genetics and predation experiments.

## Results

**Figure 1:**
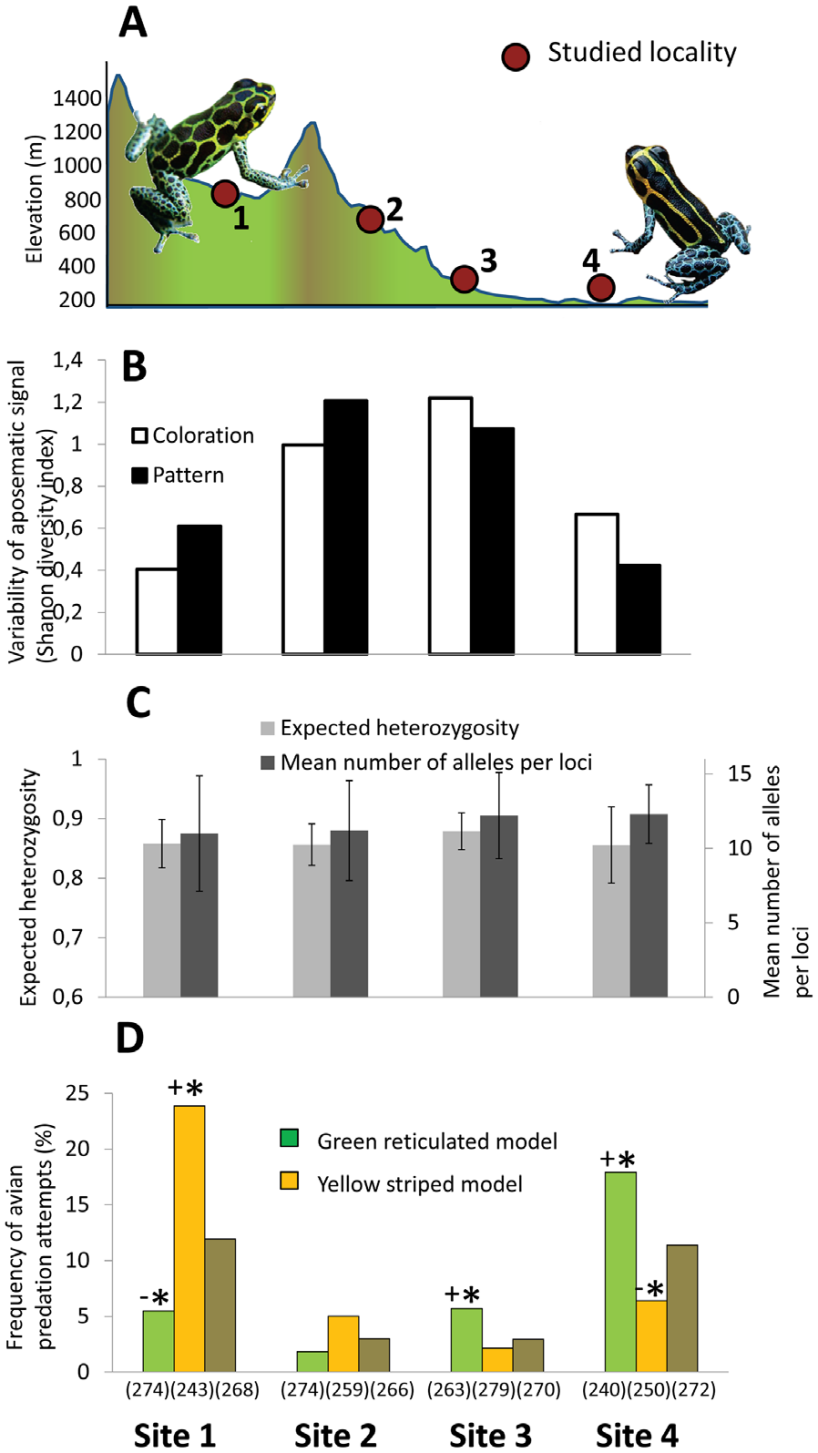
Phenotypic variability, genetic diversity and predation pressure measured in *Ranitomeya imitator* for the study sites. (A) Topographic profile of the study system with the study sites along the 18 km transect. (B) Phenotypic variation within sites of both aposematic dorsal coloration and pattern measured using the Shannon diversity index. (C) Neutral genetic variation estimated from nine nuclear microsatellites loci in terms of expected heterozygosity and mean number of alleles per loci, and their associated standard deviation. (D) Avian predation frequency on each aposematic signal in each site. Within sites Freeman-Tukey deviates identify which model phenotype suffered significantly (*) more (+) or less (−) predation attempts and are indicated above the bars, and the sample size is specified below.

*Ranitomeya imitator* is a small semi-arboreal dendrobatid frog distributed in the Peruvian department of San Martin and Loreto. While this species is principally distributed in the Amazonian lowland where it displays a vivid yellow striped aposematic signal, small phenotypically distinct populations are also found in valleys of higher elevation formed by the Cordillera Escalera [15], [30]. Four sites, each separated by *ca*. 6 km, were chosen along an altitudinal transect (Figure 1A) covering the transition in aposematic signals from one such higher elevation valley towards the Amazonian lowland. In the two most distant sites (sites 1 and 4) *R. imitator* possesses nearly fixed but distinct aposematic signals (Figure 1B; site 1: Shannon diversity index H′ = 1.069, site 4: H′ = 1.175). In the first site located in the higher elevation valley, *R. imitator* possesses a green reticulated pattern on a black background on the top of the head, dorsum and flanks. The geographical range of this phenotype is relatively small (*ca.* less than 15 km^2^), limited by the mountain ridges of the Cordillera Escalera [28]–[30]. In the lowland site (site 4), the head, dorsal and flank pattern consists of thin longitudinal yellow stripes on a black background. This phenotype is the most commonly encountered due to its large geographical range (*ca.* 5000+ km^2^) which spreads from the foot of the eastern Cordillera Escalera far into the Amazonian lowland [28]–[30].

Sites 2 and 3 were selected in between the other two sites and were located at intermediate elevations inside the only corridor appearing to connect the highland green reticulated phenotype population (site 1) to the Amazonian lowlands (site 4). These sites are characterized by a high diversity of colors and patterns (Figure 1B; H′ = 2.085 in site 2 and H′ = 2.097 in site 3) in comparison to sites 1 and 4 (W_3,132_ = 30.18, P<0.001). These phenotypes are however not randomly distributed, since the green reticulated pattern and the yellow striped pattern are predominant in sites 2 and 3 respectively. The habitat of *R. imitator* at these elevations is relatively small and fragmented due to land-use for pasture. As a result, numerous small populations connected by thin corridors of forests inhabit this region.

We first tested the null hypothesis that low (sites 1 and 4) and high (sites 2 and 3) phenotypic diversity was related to demographic processes affecting genetic diversity [37]. Genetic analyses performed on microsatellite markers revealed that the studied populations display a similar level of genetic diversity, both in term of number of alleles per locus (F_3,32_ = 1.43, P = 0.250) and heterozygosity (F_3,32_ = 0.56, P = 0.644; Figure 1C). Populations do not significantly differ from mutation-drift equilibrium, providing no evidence of recent bottleneck or expansion (P≥0.291 for both SMM and IAM). Consequently, the difference in the aposematic signal's variability is explained neither by the effective population size nor by recent demographic events inferred from neutral genetic diversity.

**Figure 2:**
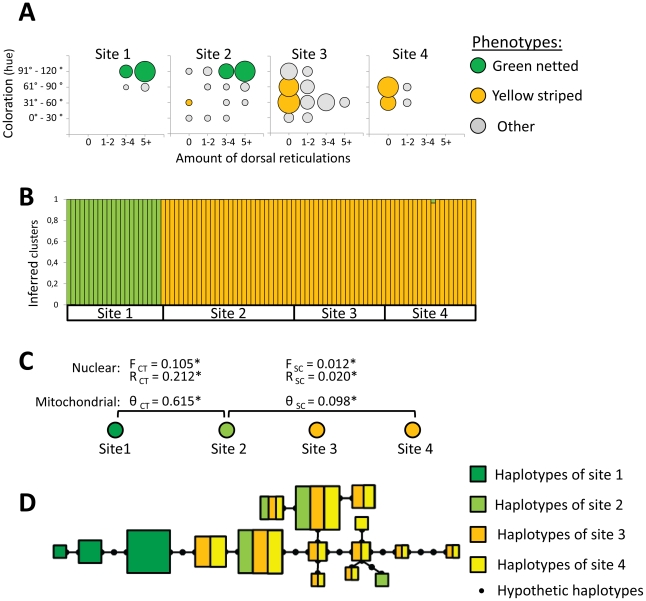
Phenotypic distribution and genetic differentiation measured in *Ranitomeya imitator* for the study sites. (A) Frequency distribution of the different aposematic phenotypic coloration and dorsal patterns found among individuals in the four study sites. (B) Individual assignment among K = 2 clusters using STRUCTURE and (C) the hierarchical partioning of genetic variance between population groups estimated from nuclear microsatellites and mitochondrial control region; * indicates a level of significance of p<0.05. (D) Minimum spanning tree for mitochondrial control region haplotypes detected in this study. Each box refers to a distinct haplotype and the size represents its relative abundance.

An alternative hypothesis to explain the higher phenotypic polymorphism observed would be that sites 2 and 3 are contact zones where hybridization occurs between the two distinct aposematic signals found in the highland and lowland, as has been documented in numerous species of *Heliconius* butterflies [12], [38]–[40]. To test this hypothesis we assessed the structure and differentiation of populations using both nuclear and mitochondrial genomes (Figures 2B, 2C, and 2D). Our results clearly show that the highland population (site 1) displaying the fixed green reticulated pattern is isolated from the other populations. The results obtained from STRUCTURE (Figure 2B) reveal that individuals of site 1 clustered together but separately from those of the other sites. These two groups identified by STRUCTURE do not share any mtDNA haplotypes (Figure 2D) and partitioning of the genetic variance between both groups measured at both the nuclear (R_CT_ = 0.212, P<0.001) and mitochondrial (θ_CT_ = 0.615, P<0.001) genome is extremely high (Figure 2C). These results are concordant with field observations that *R. imitator* is continuously distributed from the lowland (site 4) up to 500 m (site 3) into the transient zone, but absent from the Cordillera Escalera ridges or the Cainarachi River steep canyon separating these populations from the higher elevation valley (site 1). As such, current hybridization does not appear to be responsible for the phenotypic diversity.

Mitochondrial diversity of site 2 (4 haplotypes) is lower than the one of site 3 and 4 (10 and 11 haplotypes respectively) and clearly appears to be a subsample of the lower elevation sites (Figure 2D). A decrease in the number of haplotypes such as this, combined with phylogeographic studies showing that *R. imitator* originated from the Amazonian lowland [28], [30] and data on aposematic signal distribution [15], suggests that the colonization of the transient zone (sites 2 and 3) occurred recently from the yellow striped *R. imitator* which is widely distributed in the Amazonian lowlands (site 4). Tests for allelic differentiation indicated significant differences in allelic frequencies among sites 2, 3 and 4, but very low levels of differentiation (Figure 2B and 2C) in accordance with recent divergence [41]. Genetic drift is the principal process responsible for these genotypic differences between populations as it occur across the whole genome and because SPAGEDI ruled out the significant effect of mutations (P≥0.142). Moreover, even if measured on only 3 populations, genetic differentiation increases with distance and this suggests that these populations are connected according to a model of isolation by distance [42], [43].

Given similar genetic diversity and the absence of evidence for hybridization, the unusually high diversity of aposematic signals measured in the transient zone (sites 2 and 3) is likely the result of lowered predation which enables the survival of alternative phenotypes. To assess the effect of selection on aposematic signals we conducted experiments in the habitat using clay models as bait [32]. Frogs were molded and painted to resemble the two distinct fixed warning signals found in the lowlands and highlands and models representing a brown non aposematic frog were used as a predation control. These experiments (Figure 1D) confirmed the presence of significantly higher avian predation in the highland valley (site 1; FT = 4.351, P<0.05) and Amazonian lowland (site 4; FT = 3.183, P<0.05); in fact, predation was found to be approximately four times more important than in the transient zone (sites 2 and 3, FT≤−4.878, P<0.05). The low frequency of attacks on the brown non aposematic models in the transient zone strongly suggests a lower density of predators rather than a different predation regime where predators would learn to recognize different aposematic signals. In both the high predation sites (sites 1 and 4), attack frequency has previously been demonstrated to reflect the learning experience of the predator community [32], whereby the exotic aposematic phenotype (FT≥2.209, P<0.05) was more likely to be attacked by avian visual predators than the local aposematic phenotype (FT≤−2.525, P<0.05). Similarly, the most abundant phenotype in each site of the transient zone (site 2 and 3), and which is most similar to the fixed warning signal found in the neighbouring site, suffered half as many attacks despite lower predation pressures. However, these differences were not significant (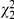
≤4.982, P≥0.082), probably due to the low statistical power caused by the low number of attacks.

## Discussion

The selection pressure on aposematic signals appears to change drastically in both intensity and direction across the studied system. At both extremities, strong stabilizing selection pressure on distinct aposematic signals are the result of predation [32]. The combination of alleles resulting in a green reticulated dorsum is maintained by predators of the highlands (site 1), whereas the yellow striped dorsum is maintained by predators of the lowlands (site 4). Any unrecognized phenotypes are rapidly eliminated, thus ensuring phenotypically monomorphic populations, an observation consistent with other aposematic systems [32]–[35], [44]–[46]. However, this predation pressure clearly decreases in the transient zone (sites 2 and 3) and coincides with an unusually high diversity of phenotypes, with many of these phenotypes not present in either the highland or the lowland sites.

In *R. imitator*, the strong phenotypic and genetic discontinuity observed between the two groups of populations (site 1 and sites 2-3-4) is concordant with isolation between both the green reticulated (site 1) and the yellow striped populations (site 4). *Ranitomeya imitator* is known to be continuously distributed from the Amazonian lowland (striped form; site 4), following the Huallaga River where transition zones with different morphs occur, all the way up to the Cainarachi Valley (green reticulated form; site 1). As such, it is likely that the yellow striped (site 4) and green reticulated *R. imitators* (site 1) represent opposite, non-communicating ends of a long distribution continuum, possibly linked by migrants through isolation by distance via the Huallaga corridor. Yet, while genes underlying the different color patterns might be exchanged between the yellow striped and green reticulated population along the Huallaga corridor, this possible gene flow cannot account for the high diversity of color patterns measured in the transient zone of the study system, as both sites 1 and 4 are far from the Huallaga transition zone.

These genetic results contrast with those observed in aposematic butterflies of the genus *Heliconius* in the very same region [34], where a similarly high diversity of phenotypes has also been documented. In both *H. erato* and *H. melpomene* butterflies, hybridization between phenotypically distinct races result in a zone where individuals display different combinations of parental race wing color patterns. In this *Heliconius* hybrid zone, while loci associated to color pattern display a strong geographic organisation due to high selection on distinct color patterns at both extremities, allele frequencies at loci unrelated to aposematism did not display differences between the phenotypically distinct populations [34]. Such difference between these systems is likely the result of different dispersal capabilities. While the ridges and canyons separating the Cainarachi Valley from the lowland do not appear to act as a barrier for butterflies that could hybridize, frogs, due to their limited dispersal capability and very small home ranges [47], likely remain isolated on each side of these barriers.

Given a similar effective population size, the negligible effect of mutations, the absence of strong demographic fluctuations and no evidence of direct hybridization, this unusually high phenotypic diversity is likely the result of the low selection pressure observed. In the absence of selection in the transient zone, all “aposematic signals” have the same fitness and the abundance of alleles linked to the genes responsible for the signal will change exclusively at random through genetic drift [19].

Interestingly, a high proportion of *R. imitator* in site 2 located in the vicinity of the highlands (site 1) display a green reticulated aposematic signal which is visually similar to the one characterizing the isolated highland population (Figure 2A). These observations are of particular interest as the *R. imitator* from this site appear to have originated from the populations inhabiting the lowland (site 4) and which display a fixed yellow striped dorsum.

Given the absence of a significant contribution of mutations, the diversity of phenotypes present in the transient zone, including the green reticulated phenotype, likely originates from epistasic combinations of alleles already present in low frequency in the lowland (site 4). If such alternative phenotypes are strongly counter selected by predators in the lowland (site 4), they are expected to survive in the absence of selection in the transient zone (site 2 and 3).

Under this scenario, the presence of a transient zone (sites 2 and 3) with low selection pressure has enabled populations originating from the monomorphic lowland populations (site 4) to randomly explore the adaptive landscape, including the neighboring highland's (site 1) adaptive peak, by drift. Although not assessed, the exploration of the adaptive landscape is likely facilitated by the presence of numerous small connected populations, as hypothesized by Wright [2], [3]. As such, a green reticulated phenotype similar to the one recognized by predators in the highland locality has appeared in these regions, and has enabled further colonization of the slopes of the Cordillera Escalera (site 2). Other phenotypes are counter selected as they approach this zone of purifying selection surrounding the highland population (Figure 1D). Because the green reticulated phenotype is avoided by experienced predators, the alleles responsible for the pattern then increases in frequency by selection and the population becomes locally adapted. Such a process, known as phenotypic advergence, between populations of the same species is not unusual, as this process is believed to be driving Batesian mimicry and sometimes occurs between species that have diverged several millions years ago [9]. This process has also been proposed to explain the phenotypic diversity of *R. imitator* ([15], but see [31]).

**Figure 3:**
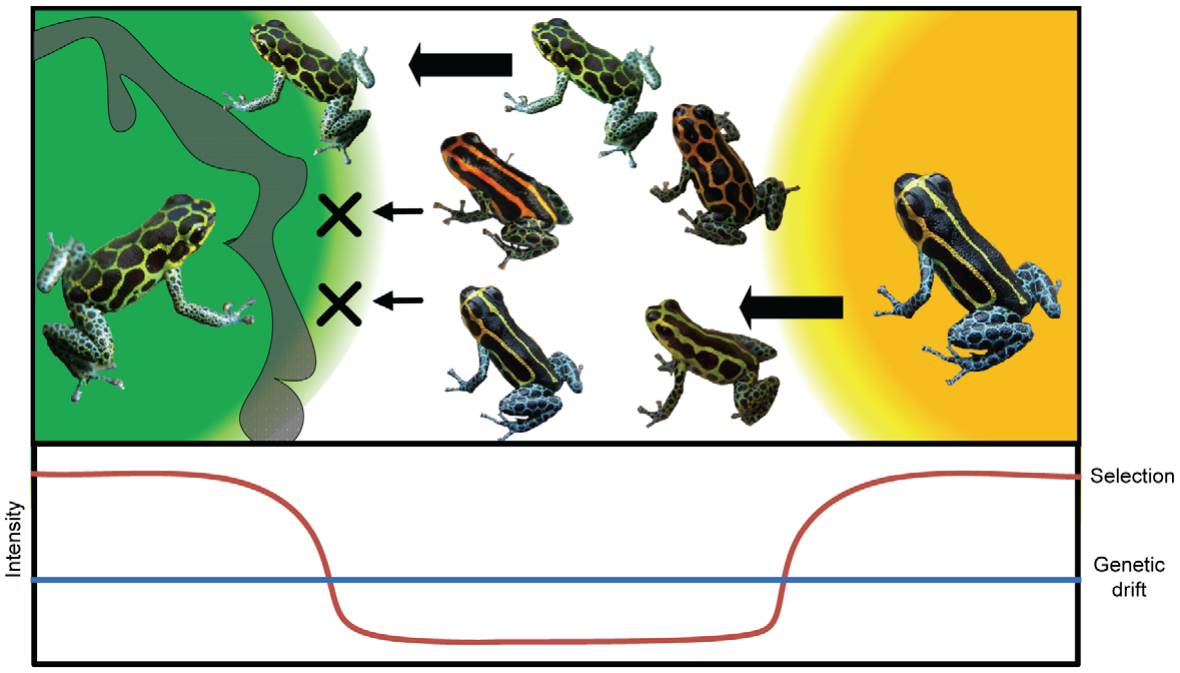
Schematic representation of the diversification process of aposematic signals in *R. imitator*. The colored areas represent zones of high predation selecting distinct phenotypes and the grey area corresponds to a migration barrier for *R. imitator*. Individuals originating from the monomorphic yellow striped lowland populations (right) colonized (arrows) the transient zone (white area). Under the lowered predation pressure prevalent in these elevations, aposematic signals diversified due to genetic drift. Among the novel signals, the green reticulated phenotype which is recognized by predators (left) enabled the further colonization of the system, while other signals are highly counter selected (X). Below the figure is a schematic representation of the relative intensity of selection compared to genetic drift.

The described scenario (Figure 3) is very similar to the two first phases proposed by Wright's SBT [2], [3]. During “Phase I”, genetic drift enables populations to explore the “adaptive landscape”, making it possible for some individuals to leave a given peak, cross valleys of low fitness and reach another “adaptive peak”. Selection then enables the population to shift to these alternative fitness peaks in “Phase II”. While the interplay of drift and selection appears to explain the differentiation of aposematic signals, this system is however clearly different from Wright's view in that the adaptive landscape is not composed of multiple peaks separated by low fitness valleys [3], [4]. Instead, the adaptive peaks are geographically structured with a major peak in a given locality. As a result, passing from one adaptive peak to the other does not imply a decrease in fitness (i.e. maladaptive combination of genes), as the selection pressures on the given characteristic are reduced, effectively flattening the adaptive landscape. Another major difference with Wright's theory is the absence of phase III which predicts that adaptation will spread to other populations and eventually to the whole species [3], [4]. This is unlikely to occur for an aposematic signal as predators learn to recognize distinct aposematic signals in different habitats, hence preventing the spread of a unique signal [33]–[35], [44], [46]. However, the novel warning signal could spread at a local scale as long as they are recognized by predators.

Three aspects of this study system enabled us to discard Fisher's LST as being the process responsible for the documented diversification. Firstly, as in other aposematic species, the selective environment changes abruptly and any phenotypes not recognized by predators are rapidly eliminated. As such, the colonization of a novel habitat where the phenotypes become exotic is improbable, thus preventing the refinement of existing adaptation as preconized by the LST [1], [7]. Under such conditions, the recognized aposematic signal must appear prior to the colonization of these habitats. Secondly, the diversification of aposematic signals in the transient zone is clearly random [48]–[52] as numerous combinations of colors and dorsal patterns are present, including many novel aposematic variants displaying features not found in either the lowland or the highland populations. Under Fisher's LST, constant directional selection would be expected to constrain the aposematic signals along the yellow striped to green reticulated phenotype continuum [5]. Finally, because the effect of mutations does not explain the differentiation between populations 2, 3 and 4 even for microsatellites loci presenting a very high rate of mutation, it appears extremely unlikely that novel mutations could explain the observed phenotypic differences [53], [54]. The absence of such mutations is in contradiction with the LST, which predicts that novel advantageous mutations will be selected and will spread within the populations [1], [4], [7].

Previous studies have shown how spatially localized selection by naïve predators can generate and maintain a mosaic of aposematic signals [32], [46], [55]. Simulations performed with multiple phenotypes randomly distributed in a given system resulted in patches of fixed but distinct phenotypes [56]. This initial distribution of multiple phenotypes is similar to the one observed in the transient zone. The present system contrasts with these simulations in that the predators (site 1) are already experienced in avoiding a given phenotype, enabling the population of site 2 to converge towards the aposematic signal present in site 1. This process is similar to the theory of advergence proposed for Müllerian mimicry [57], suggesting that if relative abundance of the two species is different, selection pressures on the rarer species to resemble the abundant form might occur. Such advergence is considered more likely when one species' aposematic signal is already established and avoided by predators before the arrival of an initially rarer second species [11], [15], [57].

In summary, we show that the interactions between drift and spatial variation in the intensity of selection are facilitating phenotypic diversification and local adaptation. Furthermore, we present compelling evidences for the process of phenotypic advergence in a Müllerian mimicry system. This study provides further evidence for the importance of drift, as emphasized by Wright's SBT, in explaining how populations have evolved in different selective environments.

## Materials and Methods

### Sampling

The field experiments were conducted in the department of San Martin (Peru) during the beginning of the rainy season (November 2009). Four sites were chosen along an altitudinal transect following the Tarapoto-Yurimaguas road: site 1 (S 06°25′17.0″ W 076°17′28.4″ alt. 514 m), site 2 (S 06°22′40.7″ W 076°17′04.4″ alt. 437 m), site 3 (S 06°19′58.4″ W 076°17′00.1″ alt. 268 m) and site 4 (S 06°17′23.0″ W 076°13′43.9″ alt. 192 m).

### Phenotypic variability

For each site a minimum of 30 *R. imitator* individuals were collected and positioned over a millimeter grid paper next to a black-grey-white card for color standardization. Each individual was then digitally photographed under standardized conditions. The individual photographs were corrected for ambient light color in reference to the black-grey-white standard in the background. The dominant hue, from the HSB (hue, saturation, and brightness) domain, was used to quantify coloration of the aposematic signal [58] using ImageJ version 1.40 g [59]. Each individual was then assigned to one of the 4 following qualitative bins based on their hue: dark orange (0°–30°), light orange (30°–60°), yellow (61°–90°) and green (91°–120°). Dorsal patterns were also characterized. The dorsal pattern in the studied sites always consisted of 3 well separated longitudinal stripes with different degrees of reticulation between them. Each photographed individual was assigned to a qualitative bin based on the amount of reticulation: no reticulation (0), slightly reticulated (1–2), reticulated (3–4) and highly reticulated (5+). Aposematic variability among each population was measured using the Shannon diversity index (H′) and comparison of variance between sites was assessed using Levene's test for equality of variances (W).

### Prediction one: Test of demographic processes

To test the null hypothesis that phenotypic diversity is related to demographic processes affecting genetic diversity, a minimum of 20 individuals per site were genotyped using nine microsatellite loci: RimiA06, RimiA07, RimiB01, RimiB02b, RimiB07b, RimiB11, RimiC05b, Rimi E02b, RimiF06 [60]. The neutral genetic diversity of each site was assessed using the expected heterozygosity and mean number of alleles par loci, measured using GENETIX [61] and compared between sites with ANOVA after verifying for the homogeneity of variances. The presence of past demographic processes was assessed under both IAM and SMM models using the BOTTLENECK [62].

### Prediction two: Test of hybridization

To test whether the higher phenotypic polymorphism observed in the transient zone is caused by hybridization between the two distinct aposematic signals found in the highland and lowland, the populations' structure and differentiation were assessed. Test of exact population differentiation was performed using GENEPOP [63] and the relevance of specific population genetic differentiation metrics (F_ST_ vs R_ST_) was tested using SPAGeDI [64]. STRUCTURE 2.3 [65] was used without prior population information to confirm population structure. Runs were performed with a burn-in length and MCMC of 100,000 generations and 10 runs were performed for K = 2 to 5. The best number of clusters K was determined *a posteriori* [66]. We also used the mitochondrial control region to assess the degree of population divergence. Primers were designed on *Oophaga pumilio* (previously called *Dendrobates pumilio*) sequences [17]. The primers 5′-AATGTATATGCCATTATC-3′ and 5′-GAAATATTATAGACCTATATC-3′ successfully amplified a segment of 274 bp and sequence alignment was performed using CLUSTALW [67]. Partition of the genetic diversity within and among populations was evaluated with Φ_ST_ and a minimum spanning network was inferred from the observed haplotypes using ARLEQUIN 3.1 [68]. Hierarchical Partitioning of genetic variance between groups previously identified using STRUCTURE was assessed using ARLEQUIN.

### Prediction three: Test of predation

To test if lowered predation enables high phenotypic diversity, 300 clay models per aposematic phenotype and 300 non aposematic brown frog models used as predation control (i.e. 900 models per site) were randomly secured on understory leaves using toothpicks in all four sites [32]. The models were left in each site 72 hours before collection. Only predation marks left by avian predators on these malleable models were defined as an attack. Models not recovered or destroyed by ants, roaches and unknown assailants were scored as missing and excluded from analysis [32]. To test variations in predation between sites, the number of attacks recorded in each site was compared using a global Chi-square test of independence on attack frequencies. When significant, the Freeman-Tukey deviates (FT) was compared to a α = 0.05 criterion (1.674), corrected for multiple comparisons using the Bonferroni method, in order to identify which site suffered significantly more or fewer predation attempts, as shown by the sign of the FT, than expected under the null hypothesis of equal attack probabilities. The same statistical approach was used to ascertain which model phenotype suffered significantly more or fewer predation attempts in each site independently (α = 0.05 criterion is 1.523).

### Accession Numbers

Sequences are deposited in the EMBL database under the following accession numbers: JF340132 - JF340141 and JQ735943 - JQ735948 and ecological data have been deposited at Dryad (DOI: 10.5061/dryad.7f4m96c3).
